# Infection prevention and control strategies for the Middle East respiratory syndrome coronavirus and outcome in Oman

**DOI:** 10.1186/2047-2994-4-S1-O58

**Published:** 2015-06-16

**Authors:** KSA Al Harthy, A Al Maani, M Elsheikh

**Affiliations:** 1 Central Department of Infection Prevention and Control, Ministry of Health HQ, Muscat, Oman

## Introduction

Middle East Respiratory Syndrome Coronavirus (MERS CoV) continues to challenge health care thru the potential to cause outbreaks with fatal outcomes. This study is a description of 5 primary cases of MERS CoV in Oman during the period, October 2013 – February 2014. The initially planned infection prevention and control (IPC) strategies and their adaptations thereafter were also described.

## Methods

The MERS CoV national taskforce recommendations on IPC aspects and the triage system were reviewed. Implemented steps and relevant weaknesses encountered during outbreaks were addressed. The investigations reports, monitoring and follow-up charts of the 5 MERS CoV reported cases were evaluated.

## Results

There were 5 cases of MERS CoV detected in Oman since 2013. The 1st case and 2nd cases were reported on 2013 and on 2015, three additional cases were diagnosed.

The 1^st^ case had 93 contacts (49% from family members and community and 51% health care workers (HCWs). High risk contacts were 18 individuals; 9 HCWs, 6 family members and 3 individuals who buried the deceased case. The screening tests of all contacts were negative and no secondary cases were identified within 2 weeks.

The 2^nd^ case had 44 contacts (61% in community and 37% HCWs). All contacts were screened except for 2 infants due to family denial. Within the 2 weeks, the screening tests were negative for all contacts and no secondary cases were identified.

The third case was admitted with lower respiratory symptoms. The wife and uncle of the index case had mild respiratory illness, both tested positive for MERS CoV. In this cluster, 114 contacts were identified (57% HCWs and 43% from community). 37 HCWs contacted with index case, 15 with wife and 10 with uncle. Initial and repeated testing in 2 weeks were negative and no further cases.

## Conclusion

The management of 5 MERS CoV cases in Oman is a success story for IPC service as no secondary cases were reported in hospital setting. The key elements in the management of the outbreak were by improving the triage in general, however, particularly on respiratory illnesses plus the enhancement of IPC precautions with relevant HCWs awareness. The large numbers of HCWs contacts were related to ambiguity of the mode of transmission and the anxiety between HCWs.

## Disclosure of interest

None declared.

